# An economic analysis of patient controlled remifentanil and epidural analgesia as pain relief in labour (RAVEL trial); a randomised controlled trial

**DOI:** 10.1371/journal.pone.0205220

**Published:** 2018-10-11

**Authors:** Liv Freeman, Johanna Middeldorp, Eline van den Akker, Martijn Oudijk, Caroline Bax, Marloes van Huizen, Celine Radder, Bianca Fong, Kitty Bloemenkamp, Albert Dahan, Michel Struys, Ben Willem Mol, Jan van Lith, Elske van den Akker-van Marle

**Affiliations:** 1 Leiden University Medical Centre, obstetrics, Leiden, the Netherlands; 2 Onze Lieve Vrouwe Gasthuis, gynaecology and obstetrics, Amsterdam, the Netherlands; 3 Amsterdam UMC, location Meibergdreef, gyneacology and obstetrics, Amsterdam, the Netherlands; 4 Amsterdam UMC, location Boelelaan, gyneacology and obstetrics, Amsterdam, the Netherlands; 5 HagaZiekenhuis, gyneacology and obstetrics, Den Haag, the Netherlands; 6 St Lucas Andreas Ziekenhuis, gyneacology and obstetrics, Amsterdam, the Netherlands; 7 Zaans Medical Centre, gyneacology and obstetrics, Amsterdam, the Netherlands; 8 Birth Centre, Wilhelmina Children Hospital, UMC Utrecht, Utrecht, the Netherlands; 9 Leiden University Medical Centre, anaesthesiology, Leiden, the Netherlands; 10 University Medical Centre Groningen, anaesthesiology, Groningen, the Netherlands; 11 The Robinson Institute, School of Paediatrics and Reproductive Health, University of Adelaide, Adelaide, Australia; 12 Leiden University Medical Centre, department of Medical Decision making, Leiden, the Netherlands; University Hospital Oldenburg, GERMANY

## Abstract

**Objective:**

To compare the costs of a strategy of patient controlled remifentanil versus epidural analgesia for pain relief in labour.

**Design:**

We performed a multicentre randomised controlled trial in 15 hospitals in the Netherlands, the RAVEL trial. Costs were analysed from a health care perspective alongside the RAVEL trial.

**Population:**

Pregnant women of intermediate to high risk beyond 32 weeks gestation who planned vaginal delivery.

**Methods:**

Women were randomised before the onset of labour, to receive either patient controlled remifentanil or epidural analgesia when pain relief was requested during labour.

**Main outcome measures:**

Primary outcome for effectiveness was satisfaction with pain relief, expressed as the area under the curve (AUC). A higher AUC represents higher satisfaction with pain relief. Here, we present an economic analysis from a health care perspective including costs from the start of labour to ten days postpartum. Health-care utilization was documented in the Case Report Forms and by administering an additional questionnaire.

**Results:**

The costs in the patient controlled remifentanil group (n = 687) and in the epidural group (n = 671) were €2900 versus €3185 respectively (mean difference of -€282 (95% CI -€611 to €47)). The (non-significant) higher costs in the epidural analgesia group could be mainly attributed to higher costs of neonatal admission.

**Conclusion:**

From an economic perspective, there is no preferential pain treatment in labouring intermediate to high risk women. Since patient controlled remifentanil is not equivalent to epidural analgesia with respect to AUC for satisfaction with pain relief we recommend epidural analgesia as the method of choice. However, if appropriately counselled on effect and side effects there is, from an economic perspective, no reason to deny women patient controlled remifentanil.

## Introduction

Epidural analgesia is considered to be the most effective analgesia during labour [1]. In recent years patient controlled remifentanil was introduced as pain relief during labour. Remifentanil is an opioid which is very suitable for administration through patient controlled analgesia (PCA) [2]. Remifentanil crosses the placenta but is rapidly metabolised by the fetus [3].

Previous studies on patient controlled remifentanil versus epidural analgesia report superior analgesia with epidural analgesia but comparable patient satisfaction [4,5]. However, these studies were small and potentially underpowered in their assessment of equivalence. We recently performed a large randomised equivalence trial to compare effects and costs of patient controlled remifentanil to epidural analgesia (RAVEL trial NTR 2551). The effectiveness study shows that women randomised to epidural analgesia were significantly more satisfied with analgesia than women randomised to remifentanil PCA with no differences in labour characteristics, neonatal parameters (Apgar score and umbilical cord pH) and maternal and neonatal admission. There were no drug related serious adverse events in the study. More women in the remifentanil group received analgesia (RR 1.3, 95% CI 1.2 to 1.5). Respiratory side effects were reported more frequently in the remifentanil group and maternal temperature was higher in the epidural group [6,7].

Only one study has been published on costs of epidural analgesia versus intravenous opioids [8]. Incremental costs for women treated with epidural analgesia were calculated based on literature review on complications and additional costs of involvement of an anaesthetist. Incremental costs were found to be $338, largely because of the increase in costs due to involvement of an anaesthesiologist.

This study reports the cost evaluation based on primary data that was performed alongside the RAVEL trial. We expected costs to be lower in the group randomised to patient controlled remifentanil as the involvement of an anaesthetist is not required.

## Material and methods

The economic analysis was performed alongside the RAVEL trial, which full design has been reported previously [6,7].

The economic analysis was performed alongside the RAVEL trial, which full design has been reported previously [6,7]. This study was approved by the Central Committee on Research Involving Human Subjects and the Medical Ethics Committee of the Leiden University Hospital (p10-240) and the Medical Ethics Committees of all participating hospitals: METC Zuidwest-Holland, METC Noord-Holland, METC Diakonessenhuis, MEC Academisch Medisch Centrum, METC VUMC, METC ZaansMC, METC St Lucas Andreas, METC UMCG, Commissie Mensgebonden Onderzoek regio Arnhem-Nijmegen, METC Maxima MC, METC Meander MC, METC Brabant, METC Twente.

Written informed consent was obtained antenatally from the women. No separate consent was obtained for neonatal information. Patient information and consent form have been added as additional information (S1 Text). The trial has been registered in the clinical trial register as NTR-2551.

In short, the RAVEL trial was a randomised controlled equivalence trial conducted from May 30^th^ 2011 until October 24^th^ 2012 in 15 centres in the Netherlands. Healthy women (American Society of Anesthesiologists’ class 1 or 2 [9]), >17 years with an intermediate to high obstetric risk who planned to deliver vaginally after 32 weeks were eligible to participate. They were randomly allocated to receive patient controlled remifentanil or epidural analgesia should they request pain relief during labour. After being informed of the study by their primary caregiver, written informed consent of the woman, was obtained at antenatal visits before onset of actual labour. There was no separate informed consent obtained for neonatal information.

Women randomised to receive remifentanil were on their request treated with remifentanil through a PCA (patient controlled analgesia) device. Women randomised to epidural analgesia were treated on their request with epidural analgesia according to local protocol.

1414 women were randomised of whom 51 women were excluded after randomisation because they delivered through elective caesarean section. There were three women lost to follow up and two women withdrew consent after randomisation; all in the epidural group. The median of the number of randomised women per hospital was 64 with an interquartile range (IQR) of 24–164. The flowchart and baseline characteristics of these women are reported elsewhere [7]. Data of all randomised women, 687 to patient controlled remifentanil and 671 to epidural analgesia were used in the costs analysis.

Economic analysis was performed from a health care perspective with a time horizon from the start of active labour until 10 days after delivery. Costs were converted to 2014 euros using the consumer price index [10].

Our published protocol stated that both effectiveness and cost-effectiveness were primary outcome measures. Satisfaction with pain relief was the primary outcome measure for effectiveness from the start of the study. As planned we performed a cost effectiveness analysis as well, taking into account the primary outcome for effectiveness. Because this was not made clear enough in the original protocol and registry it was changed in the last amended protocol. We amended the protocol after the last woman was randomised but before delivery of these women and thus before any analysis. [7].

### Resource use

Health-care utilisation was documented in the Case Report Form and by administering an additional questionnaire. Items listed in the Case Report Form were use of medication during labour, medication used in epidural analgesia and the duration of analgesia, involvement of the anaesthetist in administration of remifentanil, type of delivery (spontaneous, operative vaginal or caesarean section), repair of perineal tear in theatre, manual removal of placenta, medication used to treat postpartum haemorrhage, blood transfusion, maternal and neonatal admission (type and duration). Use of health care after discharge from the hospital was reported by participating women and measured using an additional questionnaire. Contact with general practitioner, midwife, obstetrician, paediatrician and emergency department were recorded. This paper questionnaire was handed to the woman at randomisation. If this questionnaire was in not possession of the hospital 3 weeks after delivery the woman was contacted by research nurses.

### Unit costs

For mode of delivery, operative interventions in the third stage and maternal and neonatal admission unit costs were collected from two university and two teaching hospitals. Obtained unit costs were used to calculate mean unit costs. Unit cost of maternal admission was divided into maternal ward, medium care or intensive care, for each admission. Neonatal admission was also differentiated into different levels of care, neonatal admission at the ward, medium care or high/intensive care.

Costs are obtained by multiplying resource use with their unit costs. In this study we collected information on resource use for all sites. Unit costs for labour, delivery and admission postpartum are not standardised in the Netherlands. Therefore, for these items we calculated unit costs using data from 4 hospitals.

For other unit costs, outpatient visit, visits to general practitioner, emergency department, and blood transfusion Dutch standardised prices were used [11] which were converted to 2014 euros [10]. Medication prices were obtained from the website of the pharmacotherapeutic compass [12]. Unit costs of postpartum care by community midwives were calculated using standards for yearly labour- and practice costs of midwives of the Dutch Healthcare Authority and converted to costs per hour with estimates of the yearly number of working hours of midwives of the Dutch Society for Midwifery (KNOV).

To calculate the costs of analgesia we used a bottom up approach. These costs consist of the epidural catheter and the equipment used to insert the catheter, the costs of medication used and personnel costs. Costs of the material used to insert the epidural catheter and administer medication were obtained from the purchasing department of one hospital. For personnel costs we used expert opinion of anaesthetists in two hospitals (one university and one teaching) on duration of care. For epidural analgesia this was estimated to be 30 minutes for nursing staff and 30 minutes for the anaesthetist. For patient controlled remifentanil it was advised in the study protocol to have one to one nursing for the first hour after starting analgesia. For centres where the anaesthetist was present at the start of patient controlled remifentanil their presence on the labour ward was estimated to be 20 minutes.

The amount of remifentanil used was calculated as was the amount of medication (opioids and/or local anaesthetic) used in epidural analgesia per woman based on duration of administration of analgesia. Next to equipment, material, personnel and medication costs an increment of 42% [11] of the direct costs was included for housing, depreciation and overhead. (Table 1)

**Table 1:** Cost-analyses: Units of resource use, unit costs, valuation method and volume source (2014 €).

		Unit	Unit cost	Valuation method (source)	Volume source
***Direct health care costs***					
**Admission costs**					
	Admission mother				
	hospital stay—ward	day	377	real costs*	CRF
	hospital stay—medium care	day	605	real costs*	CRF
	hospital stay—intensive care	day	1955	real costs*	CRF
	Admission child				
	hospital stay—ward	day	377	real costs*	CRF
	hospital stay—medium care	day	605	real costs*	CRF
	hospital stay—neonatal intensive care	day	1640	real costs*	CRF
	Ambulance transport	ride	292	guideline [11]	CRF
**Personnel costs**	Specialist care after discharge				
	Outpatient visit mother/neonate	visit	80	guideline [11]	AQ
	Emergency department	visit	168	guideline [11]	AQ
	General practicioner	house visit	48	guideline [11]	AQ
		visit	31	guideline [11]	AQ
		telephone contact	16	guideline [11]	AQ
	Midwife	hour	86	KNOV^	AQ
**Delivery**	*Medication during labour*				
	Oral antihypertensiva	costs per day	0.39	real costs [19]	CRF
	Oxytocin	total costs labour	0.59	real costs [19]	CRF
	Antibiotics	total costs labour	7	real costs [19]	CRF
	Fetal blood sampling	total costs labour	17	STAN trial [20]	CRF
**Pain relief during labour**	patient controlled remifentanil	procedure	10	real costs*	CRF
	epidural analgesia	procedure	19	real costs*	CRF
	Anaesthetist	hour	115	guideline [11]	CRF
	Nurse	hour	31	guideline [11]	CRF
	Equipment administration and monitoring remifentanil	procedure	6	real costs*	
	Equipment administration and monitoring epidural	procedure	15	real costs*	
**Mode of delivery**	Spontaneously	procedure	886	real costs*	CRF
	Ventouse delivery	procedure	973	real costs*	CRF
	Forcipal extraction	procedure	973	real costs*	CRF
	Caesarean section	procedure	1258	real costs*	CRF
**Third stage**	Blood transfusion				
	Red blood cells	product	224	guideline [11]	CRF
	Fresh frozen plasma	product	193	guideline [11]	CRF
	Platelets	product	541	guideline [11]	CRF
	Medication third stage				
	Oxytocin	dose per day	1	real costs [19]	CRF
	Sulprostone	dose per day	149	real costs [19]	CRF
	Balloon (Cook/Bakri)	product	176	real costs*	CRF
**Interventions post partum**	Repair perineal tear in operating theatre	procedure	1057	real costs*	CRF
	Manual removal placenta	procedure	711	real costs*	CRF
	Incomplete placenta, manual removal	procedure	682	real costs*	CRF
	Laparotomy	procedure	1518	real costs*	CRF

CRF case record form.

AQ additional questionnaire.

Source

* Real costs calculated by unit cost calculation of 2 general and 2 academic hospitals.

^ Real costs obtained through KNOV (Royal Dutch Organisation of Midwives).

### Analyses

Resource use per woman was multiplied by unit costs and total costs per woman were calculated. Mean costs differences between groups were tested using the Student’s t-test. Use of analgesia was compared using the Chi-square test. Data were analysed on an intention-to-treat basis. We used multiple imputation with SPSS to correct for missing primary outcome data [13–15]. We imputed missing AUC values for satisfaction with pain relief and pain intensity (transformed so that the distribution was approximately normal) using 20 imputed datasets. Missing values that were imputed for the cost analysis were use of oxytocin, pain relief and admission mother (all missing < 1%), admission child (missing 2%), costs of fetal scalp sampling (missing 21%), use of antibiotics during labour (missing 39%), and costs of health care after discharge (missing 56%).

Additionally we added scenario analysis post hoc to address the influence of the presence of an anaesthetist at the start of patient controlled remifentanil and to address the influence of continuous one to one nursing during administration of remifentanil. We did not plan these analysis beforehand but after the trial ended and before analysis the Dutch Heath Care Inspectorate (IGZ) initiated the development of a Standard Operating Procedure (SOP) for the administration of patient controlled remifentanil on the labour ward [16]. One of the recommendations is continuous one to one care for women treated with remifentanil. As this could influence costs we decided to perform the scenario analyses. Statistical and economic analyses were performed using SPSS version 20 (SPSS, Chicago, IL).

## Results

### Use of analgesia

**Fig 1:**
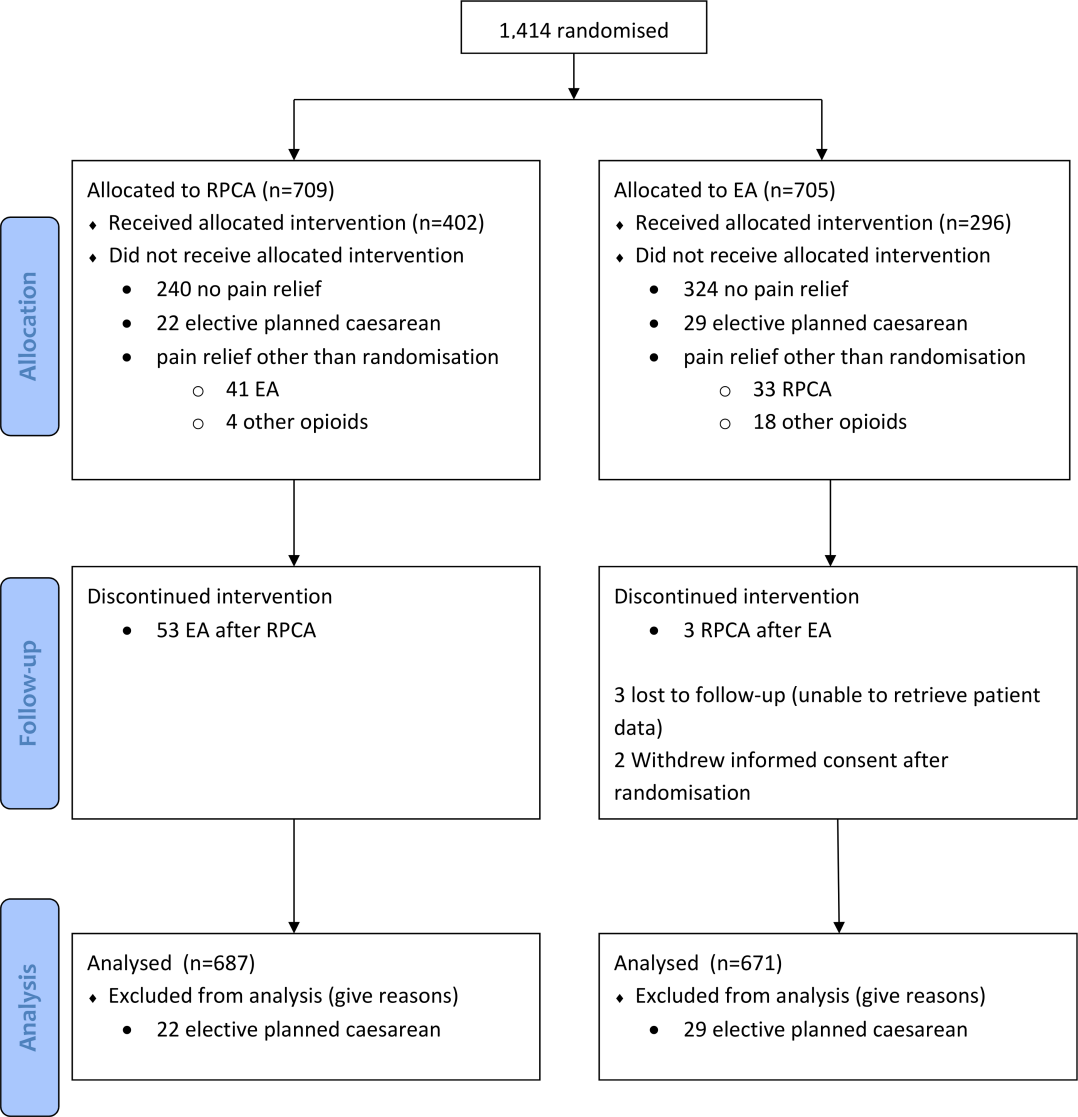
CONSORT flow chart.

In the patient controlled remifentanil group 448 women (65%) received analgesia versus 347 women (52%) in the epidural analgesia group (RR 1.3 95% CI 1.2–1.5). Of the 448 women in the remifentanil group receiving pain relief, 403 women received immediate remifentanil, of these 53 converted to epidural analgesia, 41 women received epidural analgesia and four received other opioids. Of the 347 women requesting pain relief allocated to epidural analgesia, 298 received immediate epidural analgesia (3 were also treated with patient controlled remifentanil because of insufficient pain relief), 32 were treated with patient controlled remifentanil (of whom 2 women converted to epidural analgesia after remifentanil) and 17 with other opioids. (Fig 1)

### Costs

Costs per patient are presented in Table 2. Mean costs for women randomised to patient controlled remifentanil were €2900 versus €3183 for women randomised to epidural analgesia (mean difference -€282 (95% CI -€611 to €47)). The largest part of this difference can be attributed to the higher costs of neonatal admission in the epidural group. This non-significant difference in costs for neonatal admission was -196 (95%CI -465 to 73).

**Table 2:** Costs per woman (2014 €).

	Patient controlled remifentanil		Epidural analgesia		mean difference	95% CI	p value
	mean costs pp	% patients using care	mean costs pp	% patients using care			
Analgesia							
equipment and material	6		15		-9	-10.3 to -7.3	<0.001
personnel	34		35		-0.4	-4.2 to 3.4	8.4
medication	13		5		7.7	6.4 to 9.0	<0.001
overhead*	22		23		0.6	-3.2 to 1.9	0.63
**Total analgesia**	**76**	**65**	**78**	**49**	**-2**	-10.8 to 6.6	0.64
Delivery	953	100	957	100	-5	-22 to 12	0.56
Medication during labour							
antihypertensives	0.03	9	0.03	8	0	-0.01 to 0.02	0.52
oxytocin	0.37	62	0.37	49	0	-0.04 to 0.02	0.59
antibiotics	0.35	4	0.48	9	-0.13	-0.53 to 0.27	0.53
Fetal scalp sampling	14	24	14	24	-0.17	-5.1 to 4.8	0.94
Medication third stage	7	12	7	14	-0.62	-6.8 to 5.5	0.84
Operative removal (incomplete) placenta	32	5	39	6	-7	-23 to 10	0.41
Repair of perineal tear in theatre	60	6	60	6	0.14	-25 to 26	0.99
Bloodtransfusion	11	2	19	3	-8	-19 to 3.1	0.16
**Total delivery**	**1154**		**1175**				
Maternal admission	560	62	619	63	-59	-132 to 14	0.11
Neonatal admission	1027	60	1223	63	-196	-465 to 73	0.15
10 days postpartum							
Midwife	99	96	101	96	-1	-7.7 to 5.5	0.74
General practicioner	30	48	29	48	2	-5.6 to 8.7	0.67
Obstetrician	11	5	10	6	-0.62	-8.2 to 9.4	0.89
Pediatrician	14	11	17	12	-4	-10 to 2.8	0.26
Emergency department	8	4	9	6	-2	-7.3 to 4.2	0.59
**Total postpartum**	**162**		**166**				
**Total**	**2900**		**3183**		**-282**	-611 to 47	0.09

*42% of direct costs (Hakkaart et al. 2010)

Breaking down the costs of analgesia costs for medication are higher in the remifentanil allocated group whereas costs for equipment and material are higher in the epidural allocated group (Table 2).

### Scenario analysis

Scenario analysis of the presence of an anaesthetist at the start of patient controlled remifentanil and continuous one to one care are presented in Table 3. Taking only the costs of analgesia into account, the costs of patient controlled remifentanil increase when an anaesthetist is present at the start of analgesia and increase even more with continuous one to one nursing. Only when no anaesthetist is involved in the administration of patient controlled remifentanil and there is one to one nursing for only the first hour there is a significant difference in costs of analgesia in favour of patient controlled remifentanil. In all other scenarios costs of epidural analgesia are significantly lower, resulting in even more comparable total costs between both groups.

**Table 3:** 

**Scenario analyses. Total costs.**					
	Remifentanil PCA	Epidural analgesia	difference	95% CI	p value
RAVEL trial	76	78	-2.1	-11 to 7	0.63
Scenario 1	67	77	-10	-18 to -2	0.02
Scenario 2	99	80	19	10 to 28	<0.001
Scenario 3	126	80	46	33 to 58	<0.001
Scenario 4	158	83	75	61 to 89	<0.001
**Scenario analyses. Costs of analgesia.**					
	Remifentanil PCA	Epidural analgesia	difference	95% CI	p value
RAVEL trial	2900	3183	-282	-611 to 47	0.09
Scenario 1	2892	3182	-290	-619 to 38	0.08
Scenario 2	2924	3185	-261	-590 to 68	0.12
Scenario 3	2951	3186	-235	-564 to 95	0.16
Scenario 4	2983	3189	-205	-535 to 124	0.22

Scenario 1: The anesthetist is never involved in starting patient controlled remifentanil. One to one nursing 1 hour.

Scenario 2: The anesthetist is always involved in starting patient controlled remifentanil. One to one nursing 1 hour.

Scenario 3: The anesthetist is never involved in starting patient controlled remifentanil. One to one nursing for the whole duration of administration of pain relief.

Scenario 4: The anesthetist is always involved in starting patient controlled remifentanil. One to one nursing for the whole duration of administration of pain relief.

## Discussion

### Main findings

To our knowledge this is the first study comparing the costs of patient controlled remifentanil and epidural analgesia during labour. We assessed the costs of a strategy of patient controlled remifentanil compared to epidural analgesia. Costs were analysed from a health care perspective alongside the RAVEL trial. Mean costs did not differ significantly between the two groups (mean difference -€282 (95% CI -€611 to €47), the largest difference was noted in the costs for neonatal admission. Scenario analyses show that costs of analgesia change when the anaesthetist is present and with continuous one to one nursing with patient controlled remifentanil, increasing the costs of pain relief in the remifentanil allocated group and thus increasing total costs resulting in a smaller difference between groups.

### Interpretation

We hypothesised that satisfaction with analgesia of women using patient controlled remifentanil would be equivalent to epidural analgesia. If this would be the case, women could have access to adequate analgesia with the possibility of lower costs because the presence of an anaesthetist is not required for the administration of remifentanil. Because of the low costs of both types of analgesia compared to the total costs of delivery and the post-partum period we did not show a significant difference in costs in both groups. Furthermore, the advice to provide one to one nursing of women on remifentanil in the SOP attached to the Dutch guideline will result in higher costs for remifentanil than estimated in this study, resulting in even more comparable total costs. However, latest evidence shows that one to one nursing is beneficial for all women in labour, independent of receiving analgesia or not [17]. Since this will increase costs in both groups, as shown in Table 3 scenario 3 and 4, the total difference will stay the same with a non-significant difference in costs between groups.

Costs for neonatal admission in the group randomised to epidural analgesia are almost 200 euro higher per woman, but this was not statistically significant. A possible explanation could be that mean duration of neonatal admission is 25% longer, although not statistically different, in the epidural group (mean 1.9 versus 2.5 days; p = 0.11). Also, as there are no differences in Apgar score, umbilical cord pH or reasons for admission we did not find an explanation for these higher costs. Reasons for admission did not differ between groups (S2 Table).

There were significantly more women, randomised to remifentanil, who actually requested and received analgesia. We relate this to the perception of women that remifentanil is less invasive and more easily available. Furthermore, the time between request and start of analgesia was shorter in the remifentanil PCA group, probably because the presence of an anaesthetist is not required.

### Strengths and limitations

Strength of this study is the fact that it is a large randomised controlled trial with prospective collection of data and resource use which was performed in 15 centres within the well-organised structure of the Dutch Consortium for Healthcare Evaluation and Research in Obstetrics and Gynecology. The study also has several limitations, the first being the percentage of missing data.

The reporting of missing data in trial-based economic evaluations and the methods used to handle missing data are varied and unclear. There are several ways to deal with missing data, the use of multiple imputation is valid when data are judged to be missing at random. We used multiple imputation for the primary outcome measure (satisfaction with pain relief) as well as for several economic variables because of missing values. Most missing variables were missing in less than 5%, only 3 were missing more than 5%. The variable with the most missing values was postpartum care after discharge. This variable was evaluated with an additional questionnaire, of which the response rate was 43.7%. Women that did not return the questionnaire were actively contacted by phone or at postnatal visits. Because there were no big differences between women in care postpartum reported in the questionnaires, and care postpartum in the Netherlands is standardised with three or four home visits by a community midwife and often one visit by a general practitioner, we judged that imputation would give a representative result.

Furthermore, we did not specifically ask for readmission (not in the CRF nor in the questionnaire) so we could not evaluate costs due to readmission for complications. This could potentially influence results when one group would be more prone to developing complications which would lead to admission (infection for example). However, complications were recorded in the CRF and not significantly different in both groups.

The obstetric system and uptake of analgesia in the Netherlands are different from other Western countries where many other countries have a higher uptake of analgesia. A higher uptake of analgesia could potentially result in a bigger difference in groups.

Women were randomised before start of labour and informed about the result of randomisation. While this could be a potential source of bias for our analysis for effectiveness (as was previously published [6]) this is actually a strength for economic evaluation. To be suitable for economic evaluation a trial should be pragmatic and ideally set up for measuring effectiveness (i.e. test an intervention under real life conditions) [18]. Knowing which analgesia is available when there is a need can influence if analgesia is requested, thus influencing costs. There were significantly more women, randomised to remifentanil, who actually requested and received analgesia. We relate this to the perception of women that remifentanil is less invasive and more easily available.

After careful consideration we decided not to perform a cost-effectiveness analysis since we deemed it impossible to decide what a loss of 1 point in the AUC of satisfaction with pain relief is worth in costs. So performing this cost-effectiveness analysis would not have any clinical meaning.

We stated that our multi-centre design is a strength of this study however, inter-site differences could invoke additional variability making our strength a limitation. We repeated our costs analysis using mixed-effect modelling. This resulted in marginal differences: total costs in this analysis is €-258 95% CI [-63 to 580] p = 0.12 and in our original analysis € -282 [-47 to 611] p = 0.09. [6].

## Conclusion

From an economic perspective, there is no preferential pain treatment in intermediate to high risk labouring women. Since patient controlled remifentanil is not equivalent to epidural analgesia with respect to AUC for satisfaction with pain relief in labouring women we recommend epidural analgesia to be the treatment of choice. However, if appropriately counselled on effect and side effects there is, from an economic perspective, no reason to deny women patient controlled remifentanil.

## Supporting information

S1 Fig CONSORT checklist of information to include when reporting a randomised trial. (DOC)

S1 Text Patient information and informed consent form. (DOC)

S2 Text Full study protocol. (DOC)

S1 Table Maternal and neonatal outcome. (DOC)

S2 Table Reasons for neonatal admission. (DOCX)

S1 Dataset (SAV)
