# Supplementary material for: An economic analysis of patient controlled remifentanil and epidural analgesia as pain relief in labour (RAVEL trial); a randomised controlled trial
Source: PLoS One. 2018 Oct 11;13(10):e0205220. doi: 10.1371/journal.pone.0205220 (PMC6181333; doi:10.1371/journal.pone.0205220)
Supplement: S1 Text — (DOC) [file pone.0205220.s002.doc]

**S1 Text**

**Patient information concerning the RAVEL trial**

“Remifentanil patient controlled analgesia versus epidural analgesia during labor”

This study compares two types of analgesia during labor: remifentanil patient controlled analgesia (PCA) and epidural analgesia.

You have been asked to participate in the RAVEL trial. The aim of this trial is to investigate the effectiveness and patient satisfaction of two types of pain relief during labor. It is a nationwide trial.

**Background and study objective**

There are different types of pain relief during labor. Options are:

- pethidine: an opioid which is administered though intramuscular injection (which means injected into the muscle),
- remifentanil: an opioid which can be administered intravenously by patient controlled analgesia (this means you control the amount given by pressing a button)
- epidural analgesia.

Not all pregnant women will request pain relief during labor. If you do request pain relief the options you can choose from vary depending on the hospital in which you deliver. This study investigates the effectiveness and patient satisfaction of the two most commonly used types of pain relief in the Netherlands, remifentanil PCA and epidural analgesia.

In epidural analgesia a catheter is placed in the epidural space (in the back) by an anesthesiologist. Through this catheter pain medication is continuously administered.

The epidural is very effective in reducing pain during labor. Disadvantages are that you need to stay in bed and in most cases aren’t able to urinate spontaneously (which means the bladder has to be emptied by inserting a catheter at intervals of 2-3 hours). Additionally, a percentage of women develop a fever while on epidural pain relief.

Some years ago a new drug was introduced: remifentanil. Like pethidine, this is an opioid. The difference with pethidine is that remifentanil is administered through an intravenous line by pressing a button. You give yourself a bolus dose by pressing the button, which means you control the amount of pain medication given. This method is called patient-controlled analgesia (PCA). Remifentanil is a fast-acting drug and has a short half life compared to other opiods like pethidine. Previous studies show that remifentanil gives better pain relief than pethidine and is safer for the baby. A possible disadvantage is that you might feel drowsy and need to stay in bed.

**What will happen if you decide to participate in this study?**

When you decide to participate in this study you will be assigned to receive either remifentanil PCA or epidural analgesia should you request pain relief during labor. This will be done in the outpatient department around the 32^nd^ week of pregnancy, so you will know what kind of pain relief you will get before actual labor starts.

During administration of pain medication you will be asked every hour how much pain you are experiencing (scale 0-10) and how satisfied you are with the medication given (scale 0-10). Vital parameters like temperature, blood pressure and pulse frequency will be monitored at regular intervals. Oxygen saturation will be monitored continuously by a wristband.

If you request pain relief you will be given an IV. If you are assigned to receive remifentanil the medication will be given through this IV. If you are assigned to receive epidural analgesia only fluids will be given through the IV to avoid hypotension. The anesthesiologist will then insert an epidural catheter in women assigned to receive epidural analgesia. In both groups fetal wellbeing will be monitored continuously by cardiotocography (either externally or through an internal electrode on the baby’s head).

You will be asked to fill out two questionnaires at two different times: first when you decide to participate in the trial and second six weeks after the birth of the child. Filling out these questionnaires will take about ten minutes.

**Possible risks**

Like all drugs these types of pain relief can have side effects. Most common side effects of remifentanil are drowsiness, nausea, and itching. A less common side effect is decrease of the oxygen level in your blood; this can be corrected by giving you extra oxygen by mask. With epidural analgesia you can also experience dizziness, itching and nausea. Some women develop a fever. Lowering of your blood pressure is a common side effect. This can be rapidly corrected by administration of fluids in your IV.

**Participation is voluntary**

Participation in this trial is voluntary. You can withdraw from the study at any time without this having consequences for treatment. If you receive remifentanil and you don’t have enough analgesia, you can get epidural analgesia. The same goes for epidural analgesia, should this be insufficient you can get remifentanil PCA.

If you withdraw before given medication you will receive pain relief according to hospital protocol. Your attending physician can decide to withdraw you from the study should he/she think this is in your best interest or your baby’s.

**What is going to happen to your personal data?**

We assure you that all data will be obtained in an encoded manner and handled anonymously. The information collected during the research project will be handled confidentially and there will be no access to data for people who have nothing to do with the trial. You will not find your name in scientific papers.

Sometimes you or your baby will be transferred to another hospital after birth, in this case we don’t have all the information we need at hand. We would like your permission to request your information or your baby’s in case of transfer. We need to show that you gave permission in order to obtain this information.

Sometimes new questions arise after analysing the results of a trial. These questions can in some cases be answered by contacting participants for follow up questionnaires. We would like you to state in the informed consent form if the researchers may contact you after the trial ends for follow up. This will only happen after renewed approval of the medical ethics committee.

**Ethical considerations**

This study has been approved by the board of the Leiden university medical centre (LUMC) and the National Central Committee on Research involving Human Subjects (CCMO). Specific international guidelines on this kind of trials will be followed carefully. You can read more about this in the “General brochure for human subjects (version 3, November 2008)”.

**Insurance**

Any damage to participating human subjects due to the research protocol has been insured by our hospital. Information about the insurance is documented in the insurance policy, at the end of this letter. If you feel that damage has been done by participation in this study you can contact the researchers or your gynaecologist.

**Independent physician**

We hope that the explanation in this letter about the goal and protocol of this research project is clear. You can always contact the study investigators in case of remaining questions. If you would like to hear the opinion of a gynaecologist who is not involved in this trial, you can contact dr. F Helmerhorst Tel; 071 526 2871

**What to do in case of complaints?**

You can contact the study-investigator in case of any complaints about this research project. If you would prefer to talk to someone who is not directly involved in this trial, you can contact the Klachtencommissie (complaint committee) of the LUMC, phone number 071 526 2589.

**Finally**

Please fill out and sign the informed consent form if you decide to participate in this research project. Your signature states that you have been informed about the trial (both verbally and on paper), understand the contents and know what to expect from participation in this trial. It does not imply, however, that you are committed to participate. You can end participation in the research protocol at any time without stating a reason.

This trial is coordinated and supervised by:

Mw. Dr. K.W.M. Bloemenkamp, gynaecologist/perinatologist LUMC

Mw. Dr. J.M. Middeldorp, gynaecologist/perinatologist LUMC
Mw. Drs. L.M. Freeman, investigator of the RAVEL trial, gynaecologist in training LUMC

**Informed consent form RAVEL-trial**

“Remifentanil patient controlled analgesia versus epidural analgesia during labor”

- I have read the patient information for subjects concerning the RAVEL-trial (version 1, January 2011). I have had the opportunity to ask additional questions about the research protocol. Remaining questions have been answered in an adequate way. I have had enough time to consider participation.
- I am aware of the fact that participation in the study protocol is voluntary and that I have the right to withdraw my permission at any moment without having to state a reason.
- **I agree to participate in this research project.**
- I **do / do not*** give permission to the researchers to request medical data regarding me or my baby from other hospitals (in case of transfer) if this is necessary for the trial.
- I **do / do not*** give permission to contact me for long-term follow-up in the future.
- I **do / do not*** want to be informed about the results of this study.

Telephone number:........................................................................................................
E-mail address: ………………………………………………………………………………

*****State the preferred option by a circle

Patient’s name:

Signature: Date: ____ / ____ / ____

I have given verbal and written information concerning the research project. I am willing to answer any further questions about the protocol as far as I can. Termination of participation in this study at any time will not influence regular treatment.

Name investigator:

Signature: Date: ____ / ____ / ____
