# Supplementary material for: An economic analysis of patient controlled remifentanil and epidural analgesia as pain relief in labour (RAVEL trial); a randomised controlled trial
Source: PLoS One. 2018 Oct 11;13(10):e0205220. doi: 10.1371/journal.pone.0205220 (PMC6181333; doi:10.1371/journal.pone.0205220)
Supplement: S2 Text — (DOC) [file pone.0205220.s003.doc]

RESEARCH PROTOCOL

(February 2013)

Remifentanil patient controlled analgesia (RPCA)

versus epidural analgesia (EA) during labour.

A randomized multicenter equivalence trial.

**RAVEL trial**

**PROTOCOL TITLE** ‘Remifentanil patient-controlled analgesia (RPCA) versus epidural analgesia (EA) during labor.’

| **Protocol ID** | NTR 2551 |
| --- | --- |
| **Short title** | RAVEL trial |
| **Version** | 5.0 |
| **Date** | February 2013 |
| **Coordinating investigator/project leader** | Dr. JM Middeldorp, LUMC Leiden.  *T:* 0715262896 *| F: | E:* j.m.middeldorp@lumc.nl |
| **Project leaders**  **Principal investigator**  **Project group**  **Multicenter research: per site** | Dr KWM Bloemenkamp, LUMC, Leiden Prof Dr A Dahan, LUMC, Leiden Prof Dr JMM van Lith, LUMC Leiden Dr ME van den Akker-van Marle, LUMC, Leiden  Drs LM Freeman, LUMC, Leiden  Dr M Oudijk, UMCU, Utrecht Dr E Lopriore, LUMC, Leiden Prof Dr BW Mol, AMC, Amsterdam Prof Dr MMRF Struys, UMCG, Groningen  Drs JFGM Brouns, Zaans Medisch Centrum, Zaandam  Mw drs PCM van der Salm, Meander Medisch Centrum, Amersfoort  Mw dr CM Radder, Sint Lucas Andreas Ziekenhuis, Amsterdam  Mw dr MTM Franssen, UMCG, Groningen  Dr DNM Papatsonis, Amphia Ziekenhuis, Breda  Mw drs ME van Huizen, HagaZiekenhuis, Den Haag  Dr MEA Spaanderman, UMC St Radboud, Nijmegen  Prof dr JAM van der Post, Prof dr BW Mol, AMC, Amsterdam  Mw dr DE Wijnberger, Rijnstate Ziekenhuis, Arnhem Mw dr MM Porath, Maxima Medisch Centrum, Veldhoven  Dr N Schuitemaker, Diakonessenhuis, Utrecht.  Dr E van Beek, Antoniusziekenhuis, Nieuwegein.  Dr JM Sikkema, ZGT, Almelo.  Mw dr ESA van den Akker, OLVG, Amsterdam.  Mw dr MTM Franssen, Medisch Centrum Leeuwarden, Leeuwarden  Mw dr C Radder, St Lucas Andreas, Amsterdam.  Mw Drs ME van Hoorn, VU Medisch Centrum, Amsterdam |
|  |  |
| **Sponsor (in Dutch: verrichter/opdrachtgever)** | Leids Universitair Medisch Centrum (LUMC)  Dr JM Middeldorp  Drs EJ Vos |
|  |  |
| **Independent physician(s)** | Prof Dr FM Helmerhorst  Leids Universitair Medisch Centrum  Postbus 9600  2300 RC Leiden |
|  |  |

**PROTOCOL SIGNATURE SHEET**

| **Name** | **Signature** | **Date** |
| --- | --- | --- |
| **Head of Department:**  **Prof Dr JMM van Lith, gynecologist, LUMC.** |  |  |
| **Dr JM Middeldorp, gynecologist LUMC, project leader.** |  |  |
| **Dr KWM Bloemenkamp, gynecologist LUMC, project leader.** |  |  |
| **Drs LM Freeman, AIOS gynecology/obstetrics LUMC, principal investigator.** |  |  |

**TABLE OF CONTENTS**

1. INTRODUCTION AND RATIONALE 11

2. OBJECTIVES 14

3. STUDY DESIGN 15

4. STUDY POPULATION 16

4.1 Population (base) 16

4.2 Inclusion criteria 16

4.3 Exclusion criteria 16

4.4 Sample size calculation 16

5. INVESTIGATIONAL MEDICINAL PRODUCT 18

5.1 Name and description of investigational medicinal product 18

5.2 Summary of findings from clinical and non-clinical studies 18

5.3 Summary of known and potential risks and benefits 18

5.4 Description and justification of route of administration and dosage 19

6. METHODS 20

6.1 Study parameters/endpoints 20

6.1.1 Main study parameter/endpoint 20

6.1.2 Secondary study parameters/endpoints (if applicable) 20

6.2 Randomisation, blinding and treatment allocation 21

6.3 Study procedures 21

6.4 Withdrawal of individual subjects 22

6.5 Data collection …………………………………………………………………………...22

7. SAFETY REPORTING 23

7.1 Section 10 WMO event 23

7.2 Adverse and serious adverse events 23

7.2.1 Suspected unexpected serious adverse reactions (SUSAR) 24

7.2.2 Annual safety report 24

7.3 Follow-up of adverse events 25

7.4 Data Safety Monitoring Board (DSMB) 25

8. STATISTICAL ANALYSIS 26

8.1 Descriptive statistics 26

8.2 Interim analysis 29

9. ETHICAL CONSIDERATIONS 30

9.1 Regulation statement 30

9.2 Recruitment and consent 30

9.4 Benefits and risks assessment, group relatedness 30

9.5 Compensation for injury 30

10. ADMINISTRATIVE ASPECTS AND PUBLICATION 31

10.1 Handling and storage of data and documents 31

10.2 Amendments 31

10.3 Annual progress report 31

10.4 End of study report 32

10.5 Public disclosure and publication policy 32

11. REFERENCES 34

**LIST OF ABBREVIATIONS AND RELEVANT DEFINITIONS**

| **ABR** | **ABR form, General Assessment and Registration form, is the application form that is required for submission to the accredited Ethics Committee (In Dutch, ABR = Algemene Beoordeling en Registratie)** |
| --- | --- |
| **AE** | **Adverse Event** |
| **AR**  **AUC** | **Adverse Reaction**  **Area under the curve** |
| **CA** | **Competent Authority** |
| **CCMO** | **Central Committee on Research Involving Human Subjects; in Dutch: Centrale Commissie Mensgebonden Onderzoek** |
| **CTG** | **cardiotocografie** |
| **CV** | **Curriculum Vitae** |
| **DSMB** | **Data Safety Monitoring Board** |
| **EA Epidural analgesia** | **Epidural analgesia** |
| **EU** | **European Union** |
| **EudraCT** | **European drug regulatory affairs Clinical Trials** |
| **FBS** | **fetal blood sampling** |
| **GCP** | **Good Clinical Practice** |
| **HADS** | **Hospital Anxiety and Depression Scale** |
| **IB** | **Investigator’s Brochure** |
| **IC** | **Informed Consent** |
| **IM** | **Intra muscular** |
| **IMP** | **Investigational Medicinal Product** |
| **IMPD** | **Investigational Medicinal Product Dossier** |
| **IV** | **Intra venous** |
| **MAIN** | **Morbidity Assessment Index for Newborns** |
| **METC** | **Medical research ethics committee (MREC); in Dutch: medisch ethische toetsing commissie (METC)** |
| **NACS** | **Neurologic and Adaptive Capacity Scoring system** |
| **PCA** | **Patient Controlled Analgesia** |
| **RPCA** | **Remifentanil Patient Controlled Analgesia** |
| **(S)AE** | **(Serious) Adverse Event** |
| **SPC** | **Summary of Product Characteristics (in Dutch: officiële productinfomatie IB1-tekst)** |
| **Sponsor** | **The sponsor is the party that commissions the organisation or performance of the research, for example a pharmaceutical**  **company, academic hospital, scientific organisation or investigator. A party that provides funding for a study but does not commission it is not regarded as the sponsor, but referred to as a subsidising party.** |
| **SUSAR** | **Suspected Unexpected Serious Adverse Reaction** |
| **Wbp** | **Personal Data Protection Act (in Dutch: Wet Bescherming Persoonsgevens)** |
| **WDQ** | **Wijma Delivery Expectancy/Experience Questionnaire** |
| **WMO** | **Medical Research Involving Human Subjects Act (in Dutch: Wet Medisch-wetenschappelijk Onderzoek met Mensen** |
|  |  |

|  |  |
| --- | --- |

**SUMMARY**

**Rationale:** Epidural analgesia (EA) is considered to be the most effective method of pain relief during labor and is recommended as first method of pain relief by the Dutch Societies of Gynecologists and Anesthetists. In the Netherlands its uptake by pregnant women in labor of all ethnicities is still limited (10%), compared with other western countries (40-80%), mainly as a result of non-availability due to logistic problems.

**Objective**: To assess in women with a request for pain relief during labor the cost-effectiveness of remifentanil patient controlled analgesia (RPCA) as first choice compared to EA.

**Study design:** National multicenter randomized controlled trial (RCT), in 16 centers (university hospitals, teaching and non-teaching hospitals), in cooperation with midwifery practices.

**Study population:** Pregnant women, including low risk pregnant women under care of primary care midwives, will be offered to participate in the study. Only women who have been informed before active labor has started, will be eligible.

**Intervention:** Pregnant women with a request for pain relief during labor will be randomized before active labor has started to RPCA or EA.

**Main study parameters/endpoints:** The objective of this study is to test the hypothesis that remifentanil PCA is as effective as epidural analgesia with respect to pain appreciation scores. We will look at the following outcomes: costs, pain-appreciation, patient satisfaction, pain scores (pain-intensity), maternal and neonatal side effects.

**Analysis and sample size:** The initial analysis will be performed by intention to treat. We hypothesize that there is no difference in pain appreciation scores with the two sided test (alfa=0.05, power 1-beta=0.9). In this equivalence design in each group 102 women have to be treated to exclude a potential clinical relevant difference of 10% (10 point scale, estimated SD 2.2). After correction for cross-over and non-compliance 568 patients are required. When only 50 percent of all women need actually treatment 1136 women have to be randomized.

**Economical evaluation:** For both strategies the cost of perinatal care for mother and child of delivery and the next ten days after delivery will be registered and compared.

**Nature and extent of the burden and risks associated with participation, benefit and group relatedness:** In this study two already widely used methods of pain relief during labor will be compared. No experimental medication will be used. No additional physical examination is needed for this study, nor will extra blood be taken from the subjects. Already documented risk of remifentanil PCA are maternal respiratory depression, nausea/vomiting and itching. Known risks of epidural analgesia are hypotension, increased use of oxytocin and increased risk of instrumental delivery. Maternal side effects will be part of the study outcome. After delivery participating women will be asked to fill out a questionnaire.

# INTRODUCTION AND RATIONALE

EA is considered to be the most effective method of pain relief during labor and is recommended as first method of pain relief by the Dutch Societies of Gynecologists and Anesthetists (1). In the Netherlands its uptake by pregnant women in labor of all ethnicities is still limited (10%), compared with other western countries (40-80%), mainly as a result of non-availability due to logistic problems.

This is an undesirable situation, especially since the number of women asking for pain relief during labor is increasing.

Furthermore, at present in the Netherlands, pain relief during labor is of major interest and an important topic for pregnant women, health care providers and politicians, as is pointed out in a recent publication of the multidisciplinary board on issues in obstetric management installed by the Dutch government. One of their advices is that all Dutch women in labor should have access to adequate pain relief during labor. The working party of the Dutch guideline "Pain relief during labor" recommends using RPCA only in controlled setting and recommends a large trial. Nevertheless, over 1/3 of Dutch hospitals use RPCA on labor wards, probably because the presence of an anesthetist is not required.

A large amount of studies has been conducted on the subject of pharmacological methods of pain relief during labor, such as inhalation of nitrous oxide, parenteral injection of opioids and regional analgesia by epidural and combined spinal epidural. EA is considered to be the best form of pain relief (in reducing pain-intensity) during labor (2).

There are situations in which EA is contra-indicated, i.e. patients with coagulation disorders, a common problem in daily obstetrics in preeclampsia and HELLP syndrome, or patients with skeletal disorders. In these cases im or iv opioids provide an alternative. The most commonly used opioid is im pethidine. However, its analgesic effectiveness is widely challenged (3,4,5).

Recently a new opioid, remifentanil, was introduced. Remifentanil, is a new synthetic opioid (anilidopiperidine) with direct agonist action specifically on µ-opioid receptors (6). The rapid onset and offset of the drug make remifentanil very suitable for administration via patient controlled analgesia (PCA), which can be used for analgesia during labor. Placental transfer of remifentanil does occur but appears to be rapidly metabolized, redistributed, or both. There were no adverse neonatal or maternal effects, only mild maternal sedation and respiratory changes (7). There have been multiple clinical studies on the use of remifentanil in the parturient (8-20).

A recent randomized study that compared RPCA with EA showed that in terms of pain scores (pain-intensity), EA is superior to that provided by RPCA. However, there was no difference in the pain appreciation scores between the treatments (21).

These findings were confirmed in our own pilot study that compared pain and pain appreciation scores in 20 women, randomized for RPCA or EA.

In our unpublished data (pilot study and STER study), 100 women received RPCA and only in one case medication had to be stopped because of serious side-effects (decrease in respiratory rate).

We hypothesize that RPCA is as effective in improving pain appreciation scores as EA, with lower costs and easier achievement of 24 hours availability of pain relief for women in labor.

Since we know from other studies that equality in pain appreciation scores can be anticipated, this study will, next to pain appreciation, also investigate patient satisfaction and costs. In this study patients receiving PCA remifentanil will titrate themselves to an acceptable level of pain relief by pressing a button (and receiving an analgesic bolus dose).

Equi-analgesia or equi-efficacy among the treatment groups is an essential part of the study as it is the primary end-point of analgesic treatment during labor. The definition of equi-analgesia in our study is a difference in pain appreciation (VAS score) among the two study groups of 10% or less. Preliminary data in perioperative patients on PCA opioid treatment (Dahan, oral communication) indicate that at VAS scores ranging from 4 to 8 (the expected range in our study), changes (i.e. increases) in pain score of 10% or larger will prompt an action of the patient (i.e. she will require additional pain relief by pressing the PCA button). Extrapolating these data to the current study suggests that at VAS score differences of 10% or greater clinical differences in pain appreciation may be assumed.

It is expected that economical evaluation of both strategies will show a significant reduction in costs of this innovative strategy.

Other outcome measures are pain scores, patient satisfaction, maternal and neonatal outcome and validated questionnaires.

This study considering cost effectiveness of RPCA as first choice analgesia versus EA could strongly improve the care for 180.000 women, giving birth in the Netherlands yearly (according to LVR data (2008) 30% of pregnant women under care in a hospital need pain relief during labor), by giving them access to pain relief during labor if needed, 24 hours a day.

In the guidelines of gynecologist and midwives it is recommended to discuss the opportunities of pain relief during labor antenatal (1).

An estimation of costs for RPCA versus EA shows a decrease of 64 euro per patient. The difference in costs is due to the extra costs of anesthetic staff and nurses, required when EA is given. Nationwide this could potentially save 1.2-4.6 million euro per year, depending on the percentage of women needing pain relief, varying respectively from 10-40%.

The results of the study will provide insight on whether RPCA in women in labor will reduce costs as compared to EA, assuming RPCA is equivalent to EA with respect to pain appreciation scores. At present, no clinical study has been published or undertaken to investigate this issue.

This study will be a national multicenter RCT embedded within the Dutch Obstetric Consortium, in 16 centers (university hospitals, teaching and non-teaching hospitals) in cooperation with midwifery practices . Pregnant women will be randomized to RPCA or EA, before active labor has started and thereby will be informed on the assigned method of pain relief before labor starts (as in usual care). They will be only given pain relief during labor at their request or if a medical reason should arise.

Feasibility and compliance:

An ongoing study "Differences in maternal temperature and saturation after administration of remifentanil patient controlled analgesia (RCPA) or epidural analgesia (EA) during labor; STER study" in the Leiden University Medical Centre and in a recently published study on pain relief showed that randomization of women for the proposed methods of pain relief is feasible (22).

In the Netherlands, pain relief during labor is at present of major interest and an important topic for pregnant women, health care providers and politicians, as is pointed out in a recent publication of the multidisciplinary board on issues in obstetric management installed by the Dutch government. One of their advices is all Dutch women in labor should have access to

adequate pain relief during labor (23). Furthermore, the number of referrals to the hospital because of prolonged labor and request for pain relief in increasing, which will result in more women with a need of pain relief during labor. These developments will stimulate women to participate in studies on pain relief in labor.

We estimate that for proposed study for the inclusion of 1136 women in 27 months, at least 10 hospitals are needed. The collaborating hospitals have already 24 hours availability of epidural analgesia.

# OBJECTIVES

**Primary Objective:**

The objective of this study is to test the hypothesis that remifentanil PCA is as effective as epidural analgesia with respect to pain appreciation scores.

**Secondary Objectives:**

We will also look at the following outcomes:

- Overall score of satisfaction with pain after delivery
- pain scores
- maternal and neonatal side effects and outcome
- Costs

# STUDY DESIGN

Multicentre non-blinded randomized controlled clinical trial. Time schedule will be 36 months, 3 months for preparations, 27 months for inclusions and 6 months for data analysis and economic evaluations. The study will be embedded within the Dutch Obstetric Consortium, starting in 10 centers (university hospitals, teaching and non-teaching hospitals).

Counseling and obtaining informed consent of low risk pregnant women will be done by their own primary care midwives, supported by research nurses/midwives of the Dutch Obstetric Consortium.

# STUDY POPULATION

## Population (base)

Pregnant women who have been informed before active labor has started will be eligible.

Active stage of labor is defined as presence of regular, painful uterine contractions at

regular intervals of 2-3 minutes with cervical dilation.

## Inclusion criteria

- - - - Age >18 years
      - ASA physical status I or II

## Exclusion criteria

- Drug allergy: history of hypersensitivity to opioid or local anesthetic substances
- Contra-indication for epidural analgesia

During pregnancy, after randomization, the following are reason to withdraw the parturient from the study:

- Labor before 32 weeks or after 42 weeks of gestation
- Initial maternal SpO2 of less than 95%
- Initial maternal temperature of 38C or higher.
- Prior administration of regional of opioid analgesia (during this delivery within 6 hours before request pain relief)

Absolute contra-indications for epidural analgesia are fever with evidence of systemic infection, high intracranial pressure, use of anticoagulants (coumarine derivates, LMWH prophylactic dose <12 hours, therapeutic dose <24 hours), clotting disorders with longer bleeding time, thrombocytopenia (<80). It is possible that the anesthesiologist responsible for the epidural finds other contra-indications for epidural analgesia. This will be documented in the case report form.

## Sample size calculation

The initial analysis will be performed by intention to treat. We hypothesize that there is no difference in pain appreciation scores with the two sided test, an alpha of 0.05 and 90% power. In this equivalence design in each group 102 women have to be treated to exclude a potential clinical relevant difference of 10% (10 point scale, estimated SD 2.2). Allowing for 10% and 30 % cross-over/ non-compliance in the control group and experimental group respectively, 568 patients are required. When only 50 percent of all women need actually treatment 1136 women have to be randomized. We estimate that in the group of pregnant women who are willing to participate in the study 50% will actually need pain relief. This in contrast to the general Dutch pregnant population, which is known for a low uptake of pain relief during labor.

**4.4.1** Sample size for population of low risk pregnant women.

We hypothesize that there is no difference in pain appreciation scores with the two sided test, an alpha of 0.05 and 90% power. In this equivalence design in each group 102 women have to be treated to exclude a potential clinical relevant difference of 10% (10 point scale, estimated SD 2.2).

We estimate that in the group of pregnant women who are willing to participate in the study 50% actually need pain relief. Therefore 408 women have to be randomized.

# INVESTIGATIONAL MEDICINAL PRODUCT

## Name and description of investigational medicinal product(s)

Remifentanil (Ultiva®)

Ropivacaine (Naropin®)

Sufentanil (Sufenta®)

## Summary of findings from clinical and non-clinical studies

Remifentanil, is a new synthetic opioid (anilidopiperidine) with direct agonist action specifically on mu-opioid receptors (7). The rapid onset and offset of the drug make remifentanil very suitable for administration via patient controlled analgesia (PCA).

Remifentanil is used as an analgesic agent for use during induction and/or maintenance of general anesthesia. It is also used as PCA in postoperative patients. Indications, contra-indications, farmacodynamic properties and possible side effects are described in the SmPC (Summery of Product Characteristics) of remifentanil/ Ultiva®(24).

Remifentanil PCA can be used for analgesia during labor. Placental transfer of remifentanil does occur but appears to be rapidly metabolized, redistributed, or both. There were no adverse neonatal or maternal effects, only mild maternal sedation and respiratory changes (8). There have been multiple clinical studies on the use of remifentanil in the parturient (9-21). In our unpublished data (pilot study and STER study), 100 women received RPCA and only in one case medication had to be stopped because of serious side-effects (decrease in respiratory rate).

In epidural analgesia low concentration of local anesthetic (ropivacaine) with opiates (sufentanil) is administered via continuous infusion in the epidural space. Reported maternal complications include hypotension, itching, drowsiness and fever. Also accidental punctuation of the dura which can result in post-dural headache. Epidural analgesia can also influence the course of labor (2).

## Summary of known and potential risks and benefits

Already documented risk of remifentanil PCA are maternal respiratory depression, nausea/vomiting and itching. Known risks of epidural analgesia are hypotension, increased use of oxytocin and increased risk of instrumental delivery.

## Dosages, dosage modifications and method of administration

Remifentanil patient controlled analgesia, intravenous administration; 30 microgram boluses, lockout time 3 minutes, maximum dose limit 1200 microgram/hour. It is possible to increase the bolus dosage to 40 microgram in case of insufficient pain relief, or to decrease the dose to 20 microgram in case of signs of respiratory depression or sedation.

Epidural analgesia, epidural administration according to local protocol. Suggested protocol: loading dose of 25 mg (12.5 ml ropivacaine 0.2%), continuous infusion of ropivacaine 0,1% plus sufentanil 0,5 microgram/mL.

# METHODS

## Study parameters/endpoints

### Main study parameter/endpoint

### The main outcome parameter is pain appreciation. Women will be asked to express their level of satisfaction with pain from the start of labor hourly and after start of pain relief every 15 min during the first hour and hourly after that. This will be scored on a visual analogue scale (VAS) ranging from 1 (highly dissatisfied) to 10 (highly satisfied). Total pain relief (TOTPAR) is a time weighted measure of area under the curve (AUC) or total area under the pain appreciation curve and is a summary measure that integrates serial assessments of a subject’s pain appreciation over the duration of the study.

### Secondary study parameters/endpoints

If RPCA is equivalent to EA with respect to pain appreciation the next endpoint will be the costs of both interventions. For both strategies the cost of perinatal care for mother and child, starting at the onset of labor and ending ten days after delivery, will be registered and compared (without discounting). The costs consist of costs of delivery/childbirth (course and mode of delivery), postnatal maternal care (hospitalization, outpatient visits), neonatal care (admission to NICU/neonatology ward, outpatient visits) and primary care (midwife, general practitioner, maternity care).

Maternal outcome

- Morbidity (post spinal headache, postpartum haemorrhage (>1 litre or administration of blood products), uterine rupture, eclampsia, amniotic fluid embolism, myocardial infarction, admission to ICU, other (defined as major maternal morbidity by local researchers).
- Maternal mortality
- Prolonged maternal admission. Admission in days measured from delivery of the child.
- Diagnosis during admission: endometritis, suspected infection, UTI treated with antibiotics, wound infection treated with antibiotics, obstructed bowel disease, pneumonia, trombo-embolic complications, hypertensive disorders, postpartum haemorrhage, post caesarean, pre-eclampisa/HELLP, admission due to condition child, other.

Maternal parameters

- pain scores, measured hourly from start of labor using a VAS scale
- pain appreciation scores, measured hourly from the start of labor and after start of pain relief every 15 min in the first hour and hourly after that on a VAS scale.
- satisfaction with pain, measured on a scale of 0-10 after delivery
- side effects, during administration of pain relief
  - hypotension (systolic blood pressure <90 mmHg of <25% below baseline)
  - nausea/vomiting (none, mild, moderate, severe)
  - itching (none, mild, moderate, severe)
  - respiratory depression (respiratory rate <8/min)maternal fever during labor (temperature >38°C)

Neonatal outcome

- Apgar scores ≤ 6 after 1 and 5 minutes
- Umbilical cord pH < 7.10
- Admissions to the neonatal ward
- Diagnosis during admission: neonatal meningitis/neonatal sepsis (suspected or proven with culture), prematurity, IUGR, hypoglycaemia, glucose control, IRDS, chronic lung disease, meconium aspiration, pneumothorax/mediastinum, apnea, necrotizing enterocolitis, asphyxia (pH umbilical atrey <7.0 and/or Apgar score 1 min 0-3), intraventricular haemorrhage, periventricular leukomalacia, observation due to maternal medication, admission due to condition mother, other.

### Other study parameters

Possible factors that could influence interpretation of maternal parameters during labor and neonatal outcome, also factors that could influence economical evaluation.

- Maternal status: age, race, medical history, obstetric history, parity, blood pressure, weight, information regarding labor (induction, meconium stained amniotic fluid, augmentation with oxytocin, instrumental delivery).
- Maternal medication: started before or during pregnancy, during labor.
- Condition of fetus: CTG tracing (normal, non-reassuring).

## Randomisation, blinding and treatment allocation

Randomization stratified for centre and parity will take place after informed consent. Randomization will be performed through a web-based database located in the central data collection unit in the AMC in Amsterdam. Women will be randomly allocated to receive epidural analgesia of remifentanil PCA when they request pain relief during labor. There will be no blinding, as this is not possible with these two treatment methods.

## Study procedures

Women will be asked to participate in this study before active labor has started, preferably during antenatal consult in the third trimester. They will be randomized and allocated to one of the treatment groups. If they request pain relief during labor they will be treated according to randomization with:

1. Remifentanil patient controlled analgesia; 30 microgram boluses, lockout time 3 minutes, maximum dose limit 1200 microgram/hour. Possibility of increasing or decreasing the bolus dose. When indicated EA is available.

2. Epidural analgesia; according to local protocol. When indicated RPCA is available.

There will be no additional invasive tests performed. Maternal vital parameters (temperature, heart rate, blood pressure, respiratory rate and pulse oxymetry) will be monitored non invasively at regular intervals. In the first hour after starting pain relief these parameters will be monitored with intervals of no longer than 15 minutes, after that with intervals of no longer than 1 hour. Maternal pulse oxymetry will also be monitored continuously.

Fetal heart rate and uterine activity will be measured continuously by external monitoring. When data are difficult to interpret or complications (such as meconium stained amniotic fluid, failure to progress in labor) occur, invasive monitoring by means of scalp electrode and intra-uterine pressure recording can be instituted.

Observations for known side effects will be made; respiratory depression, hypotension, nausea/vomiting and itching. Side effects will be managed by established local protocols.

Hypotension (systolic blood pressure < 90 mmHg or > 25% below baseline) will be treated with intravenous fluids, ephedrine 5 mg i.v. or phenylephrine 0.1 mg i.v. When oxygen saturation drops below 92% oxygen will be given.

Maternal pain score and pain appreciation score will be assessed using a visual analogue scale (VAS) ranging from 0-10. Women will be asked to mark the level of pain experienced during a contraction every hour starting at admission in the delivery room. Pain appreciation scores will be asked every hour, participating women will be asked to mark their level of satisfaction with analgesia. This will also be assessed using the visual analogue scale ranging from 0-10. After delivery an overall satisfaction score with pain(0-10) will be asked. Patient satisfaction will also be assessed by HADS and WDQ.

At delivery neonatal outcome including Apgar scores and cord blood gas analysis will be documented.

For economical analysis volumes of hospital care are measured prospectively alongside the clinical study in all participating centers as part of the case record form. Health resource use outside the hospital will be recorded by questionnaires filled out by the patients.

## Withdrawal of individual subjects

Subjects can leave the study at any time for any reason if they wish to do so without any consequences. The investigator can decide to withdraw a subject from the study for urgent medical reasons.

## Follow up of withdrawn subjects

Analysis will be done according to intention to treat.

# SAFETY REPORTING

## Section 10 WMO event

In accordance to section 10, subsection 1, of the WMO, the investigator will inform the subjects and the reviewing accredited METC if anything occurs, on the basis of which it appears that the disadvantages of participation may be significantly greater than was foreseen in the research proposal. The study will be suspended pending further review by the accredited METC, except insofar as suspension would jeopardise the subjects’ health. The investigator will take care that all subjects are kept informed.

## Adverse and serious adverse events

Adverse events are defined as any undesirable experience occurring to a subject during the study, whether or not considered related to the investigational product / the experimental treatment. All adverse events reported spontaneously by the subject or observed by the investiga­tor or his staff will be recorded.

A serious adverse event is any untoward medical occurrence or effect that at any dose:

- results in death;
- is life threatening (at the time of the event);

- requires hospitalisation or prolongation of existing inpatients’ hospitalisation;

- results in persistent or significant disability or incapacity;

- is a new event of the trial likely to affect the safety of the subjects, such as an unexpected outcome of an adverse reaction, lack of efficacy of an IMP used for the treatment of a life threatening disease, major safety finding from a newly completed animal study, etc.

- Serious adverse events that were defined for this study are respiratory failure requiring manual of assisted ventilation, CPR, anaphylactic shock, meningitis and epidural hematoma.

All SAEs will be reported to the accredited METC that approved the protocol, within 15 days after the sponsor has first knowledge of the serious adverse reactions.

SAEs that result in death or are life threatening should be reported expedited. The expedited reporting will occur not later than 7 days after the responsible investigator has first knowledge of the adverse reaction. This is for a preliminary report with another 8 days for completion of the report.

### Suspected unexpected serious adverse reactions (SUSAR)

Adverse reactions are all untoward and unintended responses to an investigational product related to any dose administered.

Unexpected adverse reactions are adverse reactions, of which the nature, or severity, is not consistent with the applicable product information (e.g. Investigator’s Brochure for an unapproved IMP or Summary of Product Characteristics (SPC) for an authorised medicinal product).

The sponsor will report expedited the following SUSARs to the METC:

- SUSARs that have arisen in the clinical trial that was assessed by the METC;

The remaining SUSARs are recorded in an overview list (line-listing) that will be submitted once every half year to the METC. This line-listing provides an overview of all SUSARs from the study medicine, accompanied by a brief report highlighting the main points of concern.

The expedited reporting of SUSARs through the web portal ToetsingOnline is sufficient as notification to the competent authority.

The sponsor will report expedited all SUSARs to the competent authorities in other Member States, according to the requirements of the Member States.

The expedited reporting will occur not later than 15 days after the sponsor has first knowledge of the adverse reactions. For fatal or life threatening cases the term will be maximal 7 days for a preliminary report with another 8 days for completion of the report.

### Annual safety report

In addition to the expedited reporting of SUSARs, the sponsor will submit, once a year throughout the clinical trial, a safety report to the accredited METC, competent authority, Medicine Evaluation Board and competent authorities of the concerned Member States.

This safety report consists of:

- a list of all suspected (unexpected or expected) serious adverse reactions, along with an aggregated summary table of all reported serious adverse reactions, ordered by organ system, per study;
- A report concerning the safety of the subjects, consisting of a complete safety analysis and an evaluation of the balance between the efficacy and the harmfulness of the medicine under investigation.

## Follow-up of adverse events

All adverse events will be followed until they have abated, or until a stable situation has been reached. Depending on the event, follow up may require additional tests or medical procedures as indicated, and/or referral to the general physician or a medical specialist.

## Data Safety Monitoring Board (DSMB)

The Data Safety Monitoring Committee of the Dutch Obstetrical Consortium has been approached to monitor the progress of the study and safety of participating women. This committee consists of prof. dr. J. Tijssen (clinical epidemiologist AMC), prof. dr. M. Offringa (neonatologist AMC) and prof. dr. F. Helmerhorst (gynecologist LUMC)

Serious adverse events (SAE) will be reported directly to this committee.

The advice(s) of the DSMB will be notified upon receipt by the sponsor to the METC that approved the protocol. With this notification a statement will be included indicating whether the advice will be followed.

# STATISTICAL ANALYSIS

## Descriptive statistics

- 1. Maternal age: continuous, quantitative
  2. ASA physical status: continuous, quantative (1-5)
  3. Body Mass Index: continuous, quantative
  4. Parity: categorical, quantitative: (nulliparous / multiparous)
  5. Previous cesarean delivery: categorical, quantitative (yes/no)
  6. Gestational age: continuous, quantitative
  7. Maternal origin: categorical, quantitative (Caucasian, Mediterranean, Creole, Asian)
  8. Educational level: categorical, quantitative
  9. Number of fetuses: categorical, quantitative (1-3)
  10. Fetal condition at time of request pain relief: categorical, quantitative (optimal, non reassuring, fetal tachycardia)
  11. Start of labor: categorical, quantitative (spontaneous/induction)
  12. Use of oxytocin: categorical, quantitative (yes/no)

Mode of delivery: categorical, quantitative (vaginal delivery, instrumental delivery,

cesarean section)

- 1. Indication for cesarean section/ instrumental delivery: categorical, quantitative Duration first stage of labor: continuous, quantitative
  2. Duration second stage of labor: continuous, quantitative
  3. Time from request to start of analgesia: continuous, quantitative
  4. Duration of analgesia: continuous, quantitative
  5. Conversion from epidural analgesia to RPCA: categorical, quantitative (yes/no)
  6. Conversion from RPCA to epidural analgesia: categorical, quantitative (yes/no)
  7. Type of medication used for epidural analgesia: categorical, quantitative
  8. Increases or decreases in bolus dose for RPCA: categorical, quantitative
  9. Maternal pain scores: continuous, quantitative (0-10)
  10. Maternal pain appreciation scores: continuous, quantitative (0-10)
  11. Maternal satisfaction with analgesia: continuous, quantitative (0-10)
  12. Maternal temperature: continuous, quantitative
  13. Start of intravenous broad spectrum antibiotics; quantitative (yes/no)
  14. Maternal saturation: continuous, quantitative
  15. Maternal respiratory depression: categorical, quantitative (yes/no)
  16. Oxygen administered: categorical, quantitative (yes/no)
  17. Maternal hypotension: categorical, quantitative (yes/no)
  18. Nausea/vomiting: categorical, quantitative (mild, moderate, severe)
  19. Itching: categorical, quantitative (mild, moderate, severe)
  20. Postpartum hemorrhage >1000 ml: categorical, quantitative (yes/no)
  21. Major maternal complication
  22. Post spinal headache: categorical, quantitative (yes/no)
  23. Apgar 1 and 5 minutes: continuous, quantitative
  24. Arterial umbilical cord pH: continuous, quantitative
  25. Admission to neonatal ward: categorical, quantitative (yes/no)
  26. Number of visits to/from primary health care mother: continuous, quantitative
  27. Number of visits to/from primary health care child: continuous, quantitative
  28. Number of visits to/from gynecologist: continuous, quantitative
  29. Number of visits to/from pediatrician: continuous, quantitative
  30. Duration of neonatal admission: continuous, quantitative

Duration of maternal admission: continuous, quantitative

We will report on the primary outcome measure, pain appreciation, as area under the pain appreciation curve (AUC), this as a measure for the aggregate effect of pain appreciation over a period of time (27). Total pain relief (TOTPAR) is a time weighted measure of AUC or total area under the pain appreciation curve and is a summary measure that integrates serial assessments of a subject’s pain appreciation over the duration of the study. The AUC will be analyzed from the start of active labor and for the whole duration of labor. We will also perform analysis from start of pain relief for the duration the pain relief is given. Subgroup analysis is planned for the AUC for 0-4 hours, 4-8 hours and >8 hours after the start of pain relief.

The trial is designed as an equivalence trial in which the null hypothesis

is that the difference in pain appreciation score between the two treatment groups is

greater than 10%. Preliminary data in perioperative patients on PCA opioid treatment show that changes (i.e. increases) in pain score of 10% or larger will prompt an action of the patient (i.e. she will require additional pain relief by pressing the PCA button). Extrapolating these data to the current study suggests that at VAS score differences of 10% or greater clinical differences in pain appreciation may be assumed.

The analysis of the randomised clinical trial will be by intention to treat, stratified for

centre and parity. First, the epidural and remifentanil group will be compared. Relative risks and 95% confidence intervals will be calculated for the relevant outcome measures. Categorical variables will be tested with the Chi-square test or Fisher’s exact test. Continuous variables will be tested with the Mann-Whitney U test. Time to delivery will be assessed using Kaplan-Meier analysis. Pain scores will be analyzed separately. We will report on pain during labor (area under the curve and mean pain score), worst pain during labor, pain scores at request pain relief. The analysis will be repeated on a par protocol basis. Subsequently, planned subgroup analysis will be done for nulliparous versus parous women, previous caesarean section, preterm labor (32 to 34 weeks and 34-37 weeks) and term labor (37-42 weeks), spontaneous versus induced labor, maternal educational level, maternal age (under 35 years versus over 35 years), multiple pregnancy. We will then use decision analysis to evaluate which intervention strategy, ie epidural analgesia or remifentanil is preferred in women who need analgesia during labor.

The low risk women will be included and analyzed in a separate trial. In the Dutch system with midwifery care and home births, there might be a difference in expectation of women with regard to pain during labor.

To be able to convince both primary and secondary care providers, these groups will be analyzed separately.

**Economic analysis:**

The costs consist of costs of delivery/childbirth (course and mode of delivery), postnatal maternal care (hospitalization, outpatient visits), neonatal care (admission to NICU/neonatology ward, outpatient visits) and primary care (midwife, general practitioner, maternity care).

Costs of delivery/childbirth are estimated by a detailed cost price analysis in which units of resource use (time of hospital staff, materials, equipment) are valued using cost per unit estimates comprising "true economic" costs, i.e. including shares of fixed costs and hospital overheads. Costs per units are estimated for both a teaching and a non-teaching hospital. A secondary analysis based on reimbursement fees will be performed. Other resource use (hospital days, outpatient visits and primary care) will be valued using standard prices (25).

In economic evaluations the preferred outcome measure for effectiveness is the quality adjusted life year. In obstetrics, the use of quality of life years as outcome measure might be less appropriate due to the relatively short duration of the delivery. As a result, many (cost) effectiveness studies focus on specific aspects of the delivery, such as pain relief and patient satisfaction (present proposal). These indicators are typically considered separately, but may be conflicting. In a HTA study additional to the present proposal (Development and validation of a delivery outcome measure. Principal investigator dr. M.E. van den Akker, aanvraagnummer Projectnet 24349) a delivery-specific classification system consisting of several relevant but separate domains will be developed. The domain scores will be converted into a utility based, composite numerical index.

## Interim analysis

No interim analysis will be performed. Because of the suggested design of the trial where equivalence is expected all 1136 women have to be randomized in order to achieve sufficient power. Both remifentanil PCA and epidural analgesia are widely used in the Netherlands as pain relief during labour and no adverse events have been recorded to date.

Serious Adverse Events (SAEs) and Suspected Unexpected Serious Adverse Reactions (SUSARs) will be reported to a Data Safety Monitoring Committee (DSMC). The DSMC can order to perform an interim analysis and, if indicated, terminate the trial prematurely.

# ETHICAL CONSIDERATIONS

## Regulation statement

This study will be conducted according to the principles of the Declaration of Helsinki (version 2008) and in accordance with the Medical Research Involving Human Subjects Act (WMO).

## Recruitment and consent

Patients will be informed about the study at antenatal visits, preferably in the first trimester. They may be informed by a doctor or a nurse. They will receive the patient information and an informed consent form. During their next visit they will be asked if they want to participate in the study. Signing of the informed consent form and randomization can be done on this visit. If patients require additional information a meeting with one of the research nurses can be arranged.

## Benefits and risks assessment, group relatedness

In this study two already widely used methods of pain relief during labor will be compared. Patients who do not wish to be in the study will receive pain relief according to local hospital protocol.

Already documented risk of remifentanil PCA are maternal respiratory depression, nausea/vomiting and itching. Known risks of epidural analgesia are hypotension, increased use of oxytocin and increased risk of instrumental delivery. Maternal side effects will be part of the study outcome.

Proposed study considering cost effectiveness of RPCA as first choice versus EA could strongly improve the care for 180.000 women, giving birth in the Netherlands yearly by giving them access to pain relief during labor if needed, 24 hours a day.

## Compensation for injury

Because in this study two common methods of analgesia during labor that are already widely used will be compared, it is possible that insurance for subjects participating might not be necessary. We would like to request the possibility not to have such insurance.

The Leiden university medical centre (LUMC) has an insurance which is in accordance with the legal requirements in the Netherlands (Article 7 WMO and the Measure regarding Compulsory Insurance for Clinical Research in Humans of 23th June 2003). This insurance provides cover for damage to research subjects through injury or death caused by the study.

1. € 450.000,-- (i.e. four hundred and fifty thousand Euro) for death or injury for each subject who participates in the Research;
2. € 3.500.000,-- (i.e. three million five hundred thousand Euro) for death or injury for all subjects who participate in the Research;
3. € 5.000.000,-- (i.e. five million Euro) for the total damage incurred by the organisation for all damage disclosed by scientific research for the Sponsor as ‘verrichter’ in the meaning of said Act in each year of insurance coverage.

The insurance applies to the damage that becomes apparent during the study or within 4 years after the end of the study.

# ADMINISTRATIVE ASPECTS AND PUBLICATION

## Handling and storage of data and documents

Data will be collected using a website dedicated to studies in the Dutch consortium for obstetric studies. Data monitoring will be done by research nurses in each of the participating centers. A substantial part of these research nurses is already working for the abovementioned consortium.

Patients will all receive a trial number at randomization, Data handling will be done anonymously, with the patient code only available to the local investigator. All data will be stored in a secured database on a secured computer, to which only the investigators will have access.

## Amendments

Amendments are changes made to the research after a favorable opinion by the accredited METC has been given. All amendments will be notified to the METC that gave a favorable opinion.

## Annual progress report

The sponsor/investigator will submit a summary of the progress of the trial to the accredited METC once a year. Information will be provided on the date of inclusion of the first subject, numbers of subjects included and numbers of subjects that have completed the trial, serious adverse events/ serious adverse reactions, other problems, and amendments.

## End of study report

The sponsor will notify the accredited METC and the competent authority of the end of the study within a period of 90 days. The end of the study is defined as the last patient’s last visit.

In case the study is ended prematurely, the sponsor will notify the accredited METC and the competent authority within 15 days, including the reasons for the premature termination.
Within one year after the end of the study, the investigator/sponsor will submit a final study report with the results of the study, including any publications/abstracts of the study, to the accredited METC and the Competent Authority.

## Public disclosure and publication policy

The results of the study will be submitted for publication in national and international peer reviewed medical journals. The results will be presented at national and international congresses of the obstetricians, anesthetists and midwives. Full confidentiality is granted to all study participants, as only completely anonymized data will be presented.

No major obstacles are to be foreseen in implementation as the outcome of the proposed study will lead to more specific recommendations on the policy in women with request for pain relief during labor.

The Dutch Society for Obstetrics and Gynecology (NVOG), the Dutch Society of Anesthetists (NVA) and the Dutch Society of Midwives (KNOV) will be committed to the outcomes of the study. The results of the project will be incorporated in the guidelines of the NVOG, NVA and KNOV.

The study will be performed in ten centers that are already collaborating in a consortium (http://www.studies-obsgyn.nl/index.asp). This will not only stimulate recruitment of patients, but it also implicates that all perinatal centers will be familiar with the study protocol and its result at the end of the study. This is likely to facilitate implementation of the study results, not only in the participating centers, but also in their affiliated clinics.

The Dutch Obstetric Consortium is launching a web based platform for the public to transfer the outcomes of their studies to pregnant women. Our study results can be spread along with the results of the other studies.

# REFERENCES

1. Guideline Pijnstilling tijdens de bevalling (pain relief during labour). NVOG (Dutch Society of Obstetrics and Gynecology) 2008
2. Anim-Somuah M, Smyth R, Howell C. Epidural versus non-epidural or no analgesia in labor. Cochrane Database Syst Rev. 2005;(4):CD000331
3. Olofsson C, Ekblom A, Ekman-Ordeberg G, Hjelm A, Irestedt L. Lack of analgesic effect of systemically administered morphine or pethidine on labor pain. Br J Obstet Gynaecol. 1996;103:968-72
4. Aly EE, Shilling RS. Are we willing to change? Anaesthesia 2000;55:419-420
5. Reynolds F, Crowhurst JA. Opioids in labor – No analgesic effect. The Lancet 1997;349:4-5
6. Michelsen LG, Hug CC Jr. The pharmacokinetics of remifentanil. Journal of Clinical Anesthesia 1996;8:679-682
7. Kan RE, Hughes SC, Rosen MA, Kessin C, Preston PG, Lobo EP. Intravenous remifentanil. Anesthesiology 1998;88:1467-749
8. Thurlow JA, Waterhouse P. Patient-controlled analgesia in labor using remifentanil in two parturients with platelet abnormalities. Br J Anaesth 2000;85:176-7
9. McCarroll CP, Paxton LD, Elliott P, Wilson DB. Use of remifentanil in a patient with peripartum cardiomyopathy requiring Caesarean section. Br J Anaesth 2001;86:135-8
10. Jones R, Pegrum A, Stacey RG. Patient-controlled analgesia using remifentanil in the parturients with thrombocytopenia. Anaesthesia 1999;54:461-5
11. Roelants F, De Franceschi E, Veyckemans F, Lavand'homme P. Patient-controlled intravenous analgesia using remifentanil in the parturient. Canadian Journal of Anesthesia 2001;48:175-178
12. Blair JM, Hill DA, Fee JP. Patient-controlled analgesia for labor using remifentanil: a feasibility study. Br J Anaesth 2001;87:415-420
13. Volikas I, Butwick A, Wilkinson C, Pleming A, Nicholson G. Maternal and neonatal side-effects of remifentanil patient controlled analgesia in labor. Br J of Anaesthesia 2005;95:504-509
14. Volikas, Male D. A comparison of pethidine and remifentanil patient-controlled analgesia in labor. Int Journal of Obstetric Anesthesia 2001;10:86-90
15. Thurlow JA, Laxton CH, Dick A, Waterhouse P, Sherman L, Goodman NW. Remifentanil by patient-controlled analgesia compared with intramuscular meperidine for pain relief in labor. Br J Anaesth 2002;88:374-378
16. Evron S, Glezerman M, Sadan O, Boaz M, Ezri T. Remifentanil: A novel systemic analgesic for labor pain. Anesth Analg 2005;100:233-8
17. Blair JM, Dobson GT, Hill DA, McCracken GR, Fee JP. Patient controlled analgesia for labor: a comparison of remifentanil with pethidine. Anaesthesia 2005;60:22
18. Volmanen P, Akural EI, Raudaskoski T, Alahuhta S. Remifentanil in obstetric analgesia: a dose-finding study. Anesth Analg 2002;94:913-917
19. Volmanen P, Akural E, Raudaskoski T, Ohtonen P, Alahuhta S. Comparison of remifentanil and nitrous oxide in labor analgesia. Acta Anaesthesiol Scand 2005;49:453-458
20. Balki M, Kasodekar S, Dhumne S, Bernstein P, Carvalho JC. Remifentanil patient-controlled analgesia for labor: optimizing drug delivery regimens. Can J Anesth 2007;54:626-633
21. Volmanen P, Sarvela J, Akural EI, Raudaskoski T, Korttila K, Alahuhta S. Intravenous remifentanil vs. epidural levobupivacaine with fentanyl for pain relief in early labor: a randomised, controlled, double-blinded study. Acta Anaesthesiol Scand 2008;52:249-55
22. Douma MR, Verwey RA, Kam-Endtz CE, van der Linden PD, Stienstra R. Obstetric analgesia: a comparison of patient-controlled meperidine, remifentanil, and fentanyl in labor. Br J Anaesth 2010;104:209-15.
23. Stuurgroep Zwangerschap en geboorte. Een goed begin. Veilige zorg rond zwangerschap en geboorte. December 2009.
24. Summary of Product Characteristics Ultiva®
25. Oostenbrink et al. Manual for cost analyses, methods and standard prices for economic evaluations in health care. Amstelveen, Dutch Health Insurance Executive Board. 2004.
26. Carvalho B, Zheng M, Aiono-Le Tagaloa L. Evaluation of experimental pain tests to predict labour pain and epidural analgesic consumption. Br J Anaesth 2012 nov 27.
