# Supplementary material for: An economic analysis of patient controlled remifentanil and epidural analgesia as pain relief in labour (RAVEL trial); a randomised controlled trial
Source: PLoS One. 2018 Oct 11;13(10):e0205220. doi: 10.1371/journal.pone.0205220 (PMC6181333; doi:10.1371/journal.pone.0205220)
Supplement: S1 Table — (DOC) [file pone.0205220.s004.doc]

**S1 Table**  Characteristics of labour in pregnant women allocated to patient controlled remifentanil or epidural analgesia according to intention to treat analysis**.**

**Figures are numbers (percentage) unless otherwise indicated**

|  | Remifentanil (n=687) | Epidural analgesia (n=671) | Relative risk (95% CI) | P value |
| --- | --- | --- | --- | --- |
| Median (IQR) gestational age at delivery (weeks) | 39.7 (38.3-40.7) | 39.7 (38.3-40.7) | — | 0.37 |
| Onset of labour: | | | | |
| Spontaneous | 282 (41) | 281 (42) | 0.98 (0.88 to 1.09) | 0.76 |
| Induced | 405 (59) | 390 (58) | 1.02 (0.91 to 1.32) | 0.76 |
| Requested pain relief | 447 (65) | 347 (52) | 1.32 (1.18 to 1.48) | <0.001 |
| Median (IQR) dilatation (cm) at request | 4 (3-5) | 4 (3-5) | — | 0.94 |
| Fetal condition at start pain relief (cardiotocography) (n=794): | | | | |
| Optimal | 400 (90) | 315 (91) | 0.96 (0.80 to 1.17) | 0.71 |
| Not optimal | 44 (10) | 32 (9) | — |  |
| Meconium stained amniotic fluid | 76 (11)* | 80 (12)† | 0.95 (0.80 to 1.13) | 0.57 |
| Augmentation with oxytocin | 394 (58) | 391 (58) | 0.97 (0.87 to 1.08) | 0.61 |
| >24 hours rupture of membraneS | 50 (7) | 48 (7) | 1.01 (0.83 to 1.24) | 0.92 |
| Median (IQR) time (min) from request to start analgesia | 28 (15-45) | 55 (32-80) | — | <0.001 |
| Median (IQR) duration of analgesia (min) | 236 (128-376) | 309 (181-454) | — | <0.001 |
| Median (IQR) duration second stage (min) | 20 (10-46) | 24 (10-53) | — | 0.09 |
| Mode of delivery: | | | | |
| Spontaneous | 518 (75) | 501 (75) | 1.01 (0.90 to 1.15) | 0.75 |
| Vaginal instrumental | 63 (9) | 70 (10) | 0.93 (0.77 to 1.13) | 0.45 |
| Caesarean section | 106 (15) | 100 (15) | 1.01 (0.88 to 1.17) | 0.87 |
| Postpartum haemorrhage (≥1000 mL) | 52 (8)‡ | 66 (10)§ | 0.86 (0.69 to 1.06) | 0.13 |
| Apgar score <7 at 5 min neonate 1 | 9 (1) | 15 (2) | 0.74 (0.44 to 1.25) | 0.20 |
| Neonate 1 pHa <7.10 | 22 (5)¶ | 28 (6)** | 0.86 (0.63 to 1.19) | 0.34 |
| Spinal headache | 1 (0.1)†† | 4 (0.6)‡‡ | 0.24 (0.03 to 2.18) | 0.21 |
| Major maternal complication | 2 (0.3) | 6 (0.9) | 0.33 (0.07 to 1.61) | 0.17 |
| Maternal admission | 419 (61) | 416 (62) | 0.98 (0.88 to 1.09) | 0.70 |
| Median (IQR) length of admission (days) | 1 (1-3) | 1 (1-3) | — | 0.24 |
| Neonatal admission | 390 (57) | 385 (57) | 0.99 (0.89 to 1.10) | 0.82 |
| Median (IQR) length of admission neonate 1 (days) | 1 (1-3) | 1 (1-3) | — | 0.13 |
| Median (IQR) length of admission neonate 2 (days) | 3 (2-5.75) | 4.5 (2.25-13.25) | — | 0.42 |

*3.2% (21) missing.

†4.2% (28) missing.

‡2% (14) missing.

§2.8% (19) missing.

¶28.7% (197) missing.

**28.8% (193) missing.

††5.3% (23/447) missing.

‡‡6.6% (22/347) missing.
