# Supplementary material for: An economic analysis of patient controlled remifentanil and epidural analgesia as pain relief in labour (RAVEL trial); a randomised controlled trial
Source: PLoS One. 2018 Oct 11;13(10):e0205220. doi: 10.1371/journal.pone.0205220 (PMC6181333; doi:10.1371/journal.pone.0205220)
Supplement: S2 Table — (DOCX) [file pone.0205220.s005.docx]

**S2 Table Neonatal admission**

| **neonate 1** | **Remifentanil PCA N=265** | **Epidural analgesia N=207** | **p value** |
| --- | --- | --- | --- |
| Neonatal sepsis suspicion | 0 | 0 |  |
| Neonatal sepsis proven with culture | 0 | 1 | 0.45 |
| neonatal meningitis suspicion | 20 | 25 | 0.13 |
| neonatal meningitis proven with culture | 1 | 4 | 0.17 |
| prematurity | 16 | 9 | 0.35 |
| dysmaturity | 25 | 20 | 0.93 |
| hypoglycemia | 15 | 16 | 0.45 |
| glucoseprotocol | 59 | 34 | 0.07 |
| IRDS | 1 | 2 | 0.58 |
| meconium aspiration | 2 | 0 | 0.51 |
| pneumothorax | 1 | 0 | 1.0 |
| apnea | 1 | 2 | 1.0 |
| asphyxia | 5 | 7 | 0.35 |
| observation because of maternal medication | 24 | 18 | 0.76 |
| admission due to condition mother/healthy newborn | 97 | 87 | 0.46 |
| 0.62 | 0.63 | 0.64 | 0.65 |
